# High Inter- and Intraspecific Variability in Amphidinol Content and Toxicity of *Amphidinium* Strains

**DOI:** 10.3390/md23090332

**Published:** 2025-08-22

**Authors:** Catharina Alves-de-Souza, Jannik Weber, Mathew Schmitt, Robert York, Sarah Karafas, Carmelo Tomas, Bernd Krock

**Affiliations:** 1 Departamento de Oceanografía, Facultad de Ciencias Naturales y Oceanográficas, Universidad de Concepción, Concepción 4030000, Chile; 2 Centro de Investigación Oceanográfica COPAS Coastal, Universidad de Concepción, Concepción 4030000, Chile; 3 Hochschule RheinMain, 65428 Rüsselsheim, Germany; jannik.weber10@googlemail.com; 4 Alfred Wegener Institut-Helmholtz Zentrum für Polar- und Meeresforschung, 27570 Bremerhaven, Germany; 5 Algal Resources Collection, Center for Marine Science, University of North Carolina Wilmington, 5600 Marvin K. Moss Lane, Wilmington, NC 28409, USA; matte.schmitt@gmail.com (M.S.); yorkra@uncw.edu (R.Y.); sarahkar00@gmail.com (S.K.); tomasc@uncw.edu (C.T.)

**Keywords:** amphidinol diversity, benthic dinoflagellates, UHPLC-MS/MS profiling, strain-specific bioactivity, harmful algal blooms

## Abstract

Amphidinols (AM) are a diverse group of bioactive polyketides produced by dinoflagellates of the genus *Amphidinium*, known for their hemolytic, antifungal, and cytotoxic activities. This work presents the assessment of AM profiles in a comprehensive number of strains, whose species boundaries were previously established through detailed taxonomic analysis. Using UHPLC-MS/MS, we characterized the spectrum of AM analogs in 54 *Amphidinium* strains isolated from diverse geographical locations. In addition, toxicity was assessed using brine shrimp assays, which revealed significant inter- and intraspecific variability. Despite the broad diversity in AM content, no clear correlation was observed between total AM levels and toxicity across all strains. Multivariate analysis grouped the strains into clusters distinguished by distinct AM profiles and toxicity levels, suggesting that AM production alone does not predict toxicity. Our findings highlight the complexity of *Amphidinium* bioactivity, emphasizing the influence of strain-specific factors and other bioactive compounds. This work highlights the importance of integrating chemical, genetic, and biological assessments to understand better the factors that govern toxicity in this genus, with implications for ecological studies and the monitoring of harmful dinoflagellates.

## 1. Introduction

The genus *Amphidinium* constitutes one of the most abundant groups of benthic dinoflagellates worldwide due to its abundance in marine habitats, ability to thrive in various environmental conditions, and potential for toxin production [1,2]. Inhabiting marine sandy sediments of intertidal or estuarine regions, *Amphidinium* species span tropical, subtropical, and temperate regions [3,4,5]. Certain species, such as *A. carterae*, *A. gibossum*, *A. massartii*, and *A. operculum*, have been linked to fish kills in various coastal regions and are suspected to enhance ciguatera symptoms associated with the occurrence of benthic harmful algal blooms (bHABs) [6,7,8,9]. Beyond their fish toxicity, *Amphidinium* species produce a variety of polyketides exhibiting a range of significant bioactivities (e.g., antifungal properties, cytotoxic effects, and antimicrobial activity). However, the ability to synthesize these polyketides is not consistent across the genus or even among all strains of the same species [10].

Among the polyketides produced by *Amphidinium* species, amphidinols (AMs) and closely related compounds, first reported by Satake et al. in 1991 [11], now comprise approximately 50 known analogs. Since there is no system of nomenclature for trivial names, a broad spectrum of different names exists for AMs, such as luteophanols (LP), lingshuiols (LS), symbiopolyols (SP), karatungiols (KAR), and carteraols (CAR) [12]. As all of these compounds possess the same polyketide carbon backbone with minor variations in carbon number and substitutions, all the previously mentioned variants are commonly collectively referred to as “amphidinols” [12]. In this context, it is worth noting that karlotoxins share the same polyketide backbone as AMs. Still, they are classified as a separate group because they are produced by pelagic species of the genus *Karlodinium* [13]. However, both types of compounds are natural, bioactive marine products that display similar (hemo)lytic and antifungal activity [11,14,15,16,17,18,19]. Despite the biological activities of AMs being attributed mainly to their ability to increase membrane permeability by binding to the cell lipid bilayer [20] and cell membranes [21], there are no registered cases of human intoxications linked to AMs. Such a duality, along with their structural diversity, suggests a rich potential of AMs for discovering new variations and bioactivities. Even though no toxic effects on humans are known, AMs severely affect marine biota and have the potential to disrupt marine food webs.

Given their adaptability and ease of culturing, *Amphidinium* species are widely maintained in culture collections and extensively studied for their genetic, physiological, and toxicological traits [22,23,24,25,26,27]. While the genus is easily recognized by its naked cells with small, left-deflecting epicones [28], identification at the species level is challenging due to morphologically conserved characters. Over the recent years, polyphasic approaches based on culturing, electron microscopy, and molecular sequencing [28] have enabled the establishment of species boundaries and the description of many new species within the genus [3,4,29,30,31,32,33]. However, previous efforts to characterize AM profiles have primarily focused on a few species, such as *A. carterae* and *A. klebsii*, and have mainly been based on strains without proper taxonomic characterization [18,34,35]. At the same time, as the toxicity of these strains is seldom simultaneously assessed in biological models, the putative role of AMs in the mortality of marine organisms remains unclear. This could lead to inconsistent and potentially misleading conclusions about the distribution and diversity of AMs across the genus, thereby complicating our understanding of the biosynthesis, ecological roles, and biotechnological applications of these polyketides.

In this study, we present the first comprehensive characterization of AM analogs using multiple UHPLC-MS/MS methods for the analysis in a significant number of *Amphidinium* strains, for which species boundaries have been previously established through detailed taxonomic characterization combining scanning electron microscopy (SEM) and various molecular techniques [29]. The toxicity levels of the 54 different strains were then established through the brine shrimp assay and compared to AM profiles to check for inter- and intraspecific patterns of geographical distribution. Additionally, the new AM variants detected in this study will be integrated into an existing UHPLC-MS/MS method, operating in selected reaction monitoring (SRM) mode, to quantify all known AM variants accurately.

## 2. Results and Discussion

### 2.1. Search for Amphidinols

A total of 54 *Amphidinium* strains were screened for AM variants. The taxonomic affiliation of these strains was previously fully resolved by Karafas et al. [29] using a combination of electron microscopy and molecular analyses (see Table S1 for details on the origin and culture conditions of the strains).

Amphidinols (AM) are large polyketides produced by marine dinoflagellates, typically with molecular weights exceeding 1000 Da. Their substantial size allows for extensive modifications of the structural backbone, resulting in a potentially high number of variants [17]. To date, ~50 AM variants have been identified and characterized to varying degrees. Some of their structures have been fully elucidated using nuclear magnetic resonance spectroscopy (NMR) [e.g., [36]], while others have been characterized primarily through mass spectrometry techniques and/or functional assays [e.g., [12]]. Although NMR remains the gold standard in natural product chemistry, it requires purified compounds in the lower milligram (mg) range—quantities that are difficult to obtain without advanced preparative facilities—and misidentification has occurred even in well-equipped labs.

In contrast, mass spectrometry (MS), while providing less structural detail than NMR, offers several practical advantages. MS can be performed at the ultra trace level (i.e., about eight orders of magnitude lower than NMR), does not require compound isolation or purification, and can analyze complex mixtures such as crude microalgal extracts. Its high sensitivity and minimal sample preparation make MS an ideal tool for exploring novel compounds. This is especially true for AMs, which exhibit two features well-suited to MS analysis: (1) a characteristic cleavage site between two vicinal hydroxyl groups in a highly conserved structural region (C1/C1’ cleavage) [12], and (2) relatively low variability in the lipophilic arm of the molecule. This arm is released as a neutral fragment following the preferred C1/C1’ cleavage, resulting in a limited set of predictable neutral losses that can be specifically targeted using neutral loss (NL) scans in tandem mass spectrometry.

Taking into account the mass spectrometric characteristics of AM, we selected an approach using three different LC-MS/MS operation modes for the exploration of novel AM variants [4]: (1) the most specific and sensitive selected reaction monitoring (SRM) mode (Table S2), which allows for the detection of target molecules of known variants, (2) the neutral loss (NL) mode for scanning for characteristic neutral AM fragments, and (3) the relatively unspecific full scan (FS) mode covering the typical mass range of AM, i.e., *m*/*z* 1000 to 1800. Positive hits detected by SRM were based solely on the defined transitions implemented in the method, and further confirmation was achieved by recording the collision-induced dissociation (CID) spectra of all detected candidate masses. As electrospray ionization (ESI) predominantly forms sodium adducts of AMs [12], which are known to stabilize polyketides, this results in limited fragmentation and CID spectra with only a few fragments [37]. This characteristic enables the rapid recognition of typical fragmentation patterns associated with AMs.

To detect potentially novel AMs, each strain was analyzed using 14 NL experiments listed in Table S3. Of these, 13 experiments produced peaks with the corresponding Q1-Masses (Table S3) in ten of the 54 strains. Additionally, FS measurements (Table S4) identified 35 additional Q1-Masses across 26 individual strains that were not detected with the NL experiments.

### 2.2. New Amphidinols

**Figure 1:**
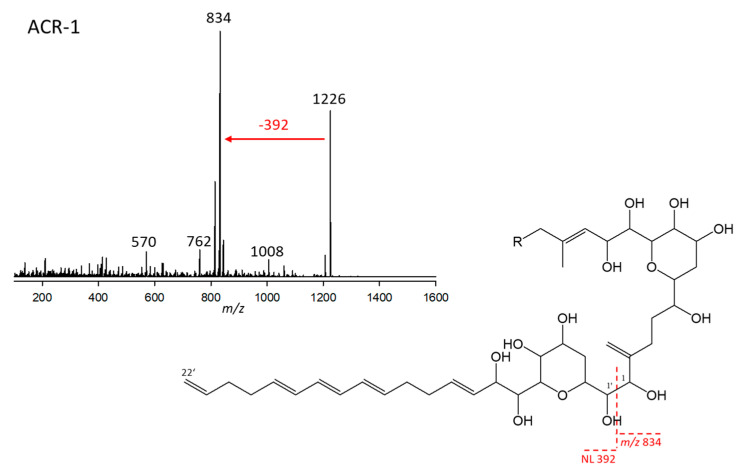
Collision-induced dissociation (CID) spectrum of ARC-1 (*m*/*z* 1226; 3.95 min) of strain *Amphidinium carterae* ARC101 (upper left, red arrow indicating the NL of the lipophilic arm) and proposed structural element based on patterns of AM fragmentation [12] containing the lipophilic arm (392 Da) and the conserved AM B-ring core structure (lower right). The dashed red line shows the cleavage site between the hydrophilic arm starting at C1 and forming the fragment *m*/*z* 834 and the neutral lipophilic arm (C1’–C22’) with 392 Da.

**Figure 2:**
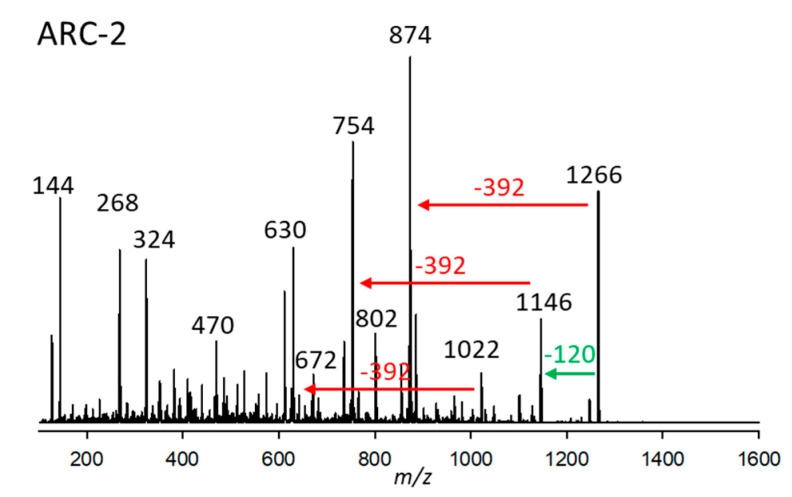
Collision-induced dissociation (CID) spectrum of ARC-2 (*m*/*z* 1266, 2.97 min) of strain *Amphidinium carterae* ARC383. The green arrow depicts the neutral loss of NaHSO_4_ (120 Da), forming the fragment *m*/*z* 1146, and the red arrows show the neutral losses of 392 Da, indicative of the lipophilic arm (C1’–C22’) of ARC-2.

**Figure 3:**
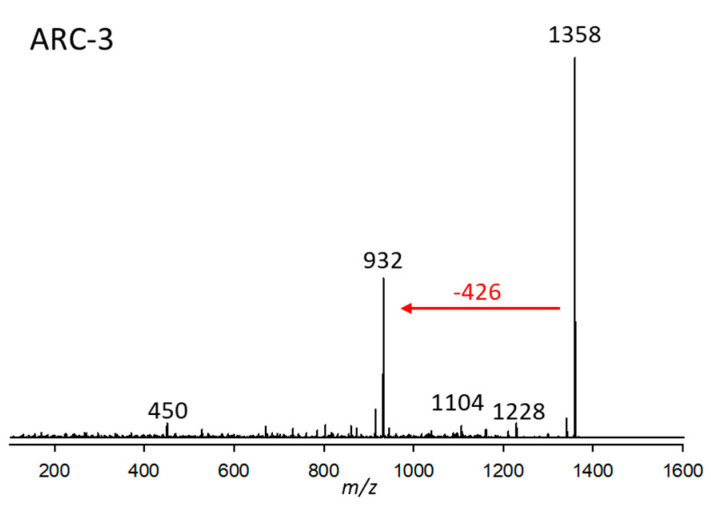
Collision-induced dissociation (CID) spectrum of ARC-3 (*m*/*z* 1358, 2.90 min) from strain *Amphidinium carterae* CCMP2100. The red arrow shows the neutral loss of 426 Da, indicative of the lipophilic arm (C1’–C22’) of ARC-3.

**Figure 4:**
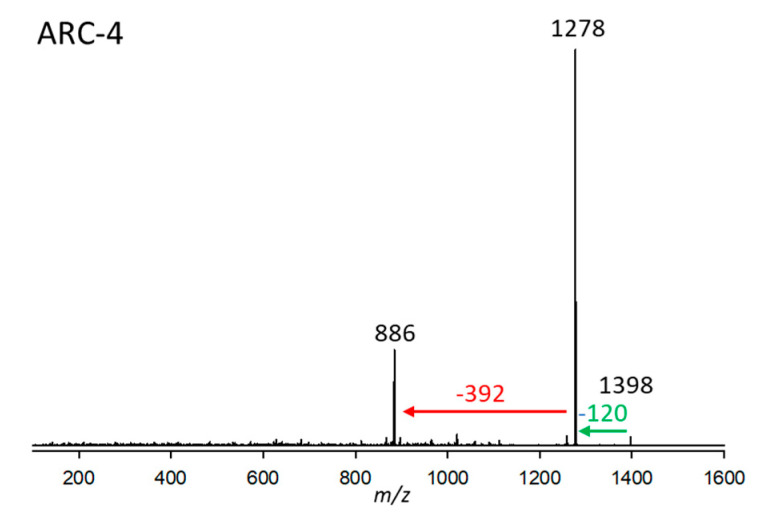
Collision-induced dissociation (CID) spectrum of ARC-4 (*m*/*z* 1398, 2.93 min) from strain *A. carterae* ARC410. The red arrow shows the neutral loss of 392 Da, indicative of the lipophilic arm (C1’–C22’) of ARC-4, while the green arrow shows the neutral loss of NaHSO_4_ (120 Da), forming the fragment *m*/*z* 1278.

Based on the described approach, 41 AMs were detected in 24 of the 54 analyzed *Amphidinium* strains, out of which eight were confirmed as novel AMs and operationally named ACR-1 to ACR-8 (Table 1). As mentioned above, the structural information of the CID spectra is limited, especially in the case of AMs, since their CID spectra display only a few fragments. The fragment-poor CID spectra of AM prevent a detailed structural characterization by mass spectrometry. However, sulfated and non-sulfated AMs can easily be distinguished by an NL of 120 Da (NaSCO_4_ [12]), indicative of sulfation (Figure 1 vs. Figure 2, and Figure 3 vs. Figure 4). Furthermore, its identity can be deduced from those of structurally known AM due to the high conservation of the lipophilic arm of AM, the fact that the C1/C1’ bond is the weakest of all known AM, and the neutral loss of the lipophilic C1’–Cn’ fragment is observed in all AM CID spectra. Nevertheless, the complete and exact chemical structures of the newly detected AM variants in this work still need to be elucidated by NMR spectroscopy in the future.

#### 2.2.1. Variant ARC-1

ARC-1 was detected in *A*. *carterae* (strains ARC98, ARC99, ARC100, ARC101) *A*. *tomasii* (strain ARC90), and *A*. *massartii* (strain CCMP1741). This variant had a retention time of 3.89 min and a pseudo-molecular mass (Na adduct) of *m*/*z* 1226. This ion fragmented into *m*/*z* 834 at the typical AM C-1’/C-1 cleavage site, resulting from the neutral loss of the lipophilic arm starting at C-1’ to C22’ with the mass of 392 Da. The 834 Da fragment created by the C-1’/C-1 cleavage was the base peak of the spectrum, indicating the LP-D type [12] of the hydrophilic arm of ARC-1. Furthermore, ARC-1 shares the neutral of 392 Da with LS-A, indicating the same lipophilic arm of both compounds (Figure 1).

#### 2.2.2. Variant ARC-2

The substance named ARC-2, present in *A*. *carterae* (strains ARC195, ARC383, ARC413), was detected at 2.97 min with a pseudo-molecular mass of *m*/*z* 1266. Its CID (Figure 2) spectrum is characterized by three losses of 392 Da from *m*/*z* 1266, 1146, and 1022, the latter being fragments formed by cleavages of the hydrophilic arm (C1-Cn) of the molecule. The loss of 392 Da indicates that ARC-1 and ARC-2 share the same lipophilic arm (C1’–Cn’) (Figure 2). Furthermore, the loss of 120 Da suggests the presence of a sulfation in the molecule, which AMs usually cleave off as NaHSO_4_.

#### 2.2.3. Variant ARC-3

ARC-3 was detected in strain *A. carterae* CCMP2100 at a retention time of 2.90 min with a pseudo-molecular mass of *m*/*z* 1358 (Figure 3). The only major fragment in its CID spectrum was *m*/*z* 932, resulting from the elimination of the neutral 426 Da fragment. This fragment, in comparison to the 392 Da fragment, is characterized by two additional hydroxylations and one double bond less.

#### 2.2.4. Variant ARC-4

ARC-4 from strain *A. carterae* ARC410 had a retention time of 2.93 min and a pseudo-molecular mass of *m*/*z* 1398. Its CID (Figure 4), like the one in ARC-3 (Figure 3), displays very little fragmentation. Besides the very low-abundant pseudo-molecular ion at *m*/*z* 1398, there is a loss of 120 Da (NaHSO_4_), resulting in *m*/*z* 1278, which indicates the presence of a sulfatation of the molecule. The resulting fragment *m*/*z* 1278 in turn eliminates the neutral fragment of 392 Da. This neutral fragment, like in the case of ARC-1 (Figure 1) and ARC-2 (Figure 2), represents the lipophilic arm (C1’–C22’) of ARC-4.

#### 2.2.5. Variant ARC-5

**Figure 5:**
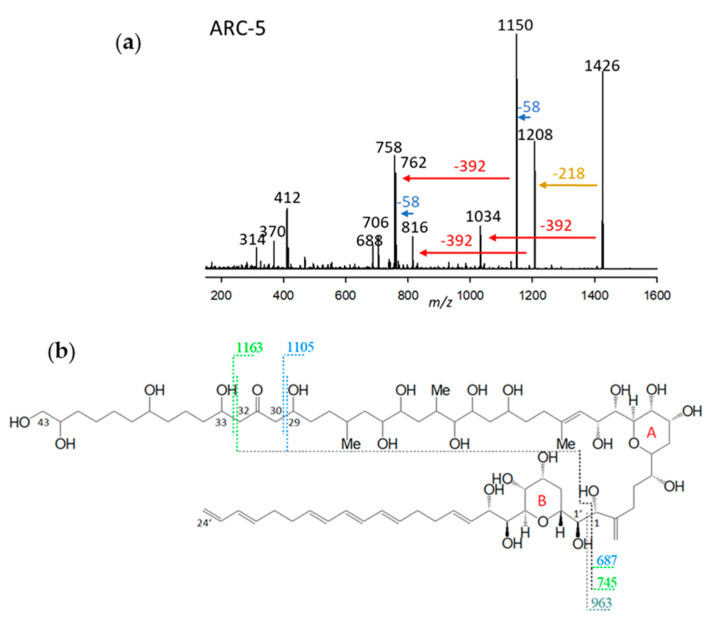
(**a**) Collision-induced dissociation (CID) spectrum of ARC-5 (*m*/*z* 1426). The red arrow shows the neutral loss of 392 Da, indicative of the lipophilic arm (C1’–C22’) of ARC-5. In contrast, the blue arrow indicates the mass difference of 58 Da, indicative of a di-γ-hydroxy-carbonyl function of ARC-5. The brown arrow shows the elimination of the structural element between the end of the hydrophilic arm and the inner γ-hydroxy function. (**b**) Chemical structure of AM-18 [37]. The fragment *m*/*z* 963 results from the C1/C1’ cleavage and elimination of the lipophilic arm (C1’–C24) (grey dashed line). The fragments *m*/*z* 687 and *m*/*z* 745 are formed by the subsequent C29/30 (blue/black dashed line) and C32/33 (green/black dashed lines) cleavages. These cleavages occur in addition to the C1/C1’ cleavage, resulting in the fragments *m*/*z* 1105 and *m*/*z* 1163, respectively. These cleavages are favored by a six-membered transition state that includes the central carbonyl function and one or the other lateral hydroxyl group, and always result in a 58 Da mass difference between fragments of the left and right cleavage sites.

ARC-5, detected in *A. carterae* (strains ARC411, ARC412), had a retention time of 3.91 min and a mass of 1426 Da (Figure 5a). The CID scan revealed a similar fragmentation pattern as AM-A, AM-B, AM-18, and AM-19 [12], which are characterized by a 29,33-di-hydroxy-31-carbonyl group (carbon numbering referring to AM-18) (Figure 5b). The di-γ-hydroxy-carbonyl function favors the cleavage of both vicinal bonds of the carbonyl group, resulting in fragments with a 58 Da mass difference via the formation of a six-membered transition state including the carbonyl and one hydroxyl group. For this reason, the occurrence of a 58 Da mass difference in AM spectra (Figure 5a) is clear evidence of the presence of a di-γ-hydroxy-carbonyl function in the molecule. The mass differences of 392 Da, which are also observed in the CID spectrum lingshuiol-A (LS-A) [12], indicate a conserved lipophilic arm (C1’–C22’) of LS-A and ARC-5. In addition, AM-18 and ARC-5 share the elimination of the neutral fragment 218 Da (Figure 5a) from the respective pseudo-molecular ions to the first fragment, which is indicative of a conserved structure between the carbonyl group and the end of the hydrophilic part (Figure 5b).

In summary, ARC-5 is characterized by the lipophilic arm shared with LS-A and a di-γ-hydroxy-carbonyl function and the structural element from the carbonyl group to the end of the hydrophilic arm shared with AM-18. All structural differences between AM-18 and ARC-5, except for the 2-carbon-atom-shorter lipophilic arm, apparently are located between C1 and C29 of ARC-5 (carbon numbering referred to AM-18).

#### 2.2.6. Variant ARC-6

**Figure 6:**
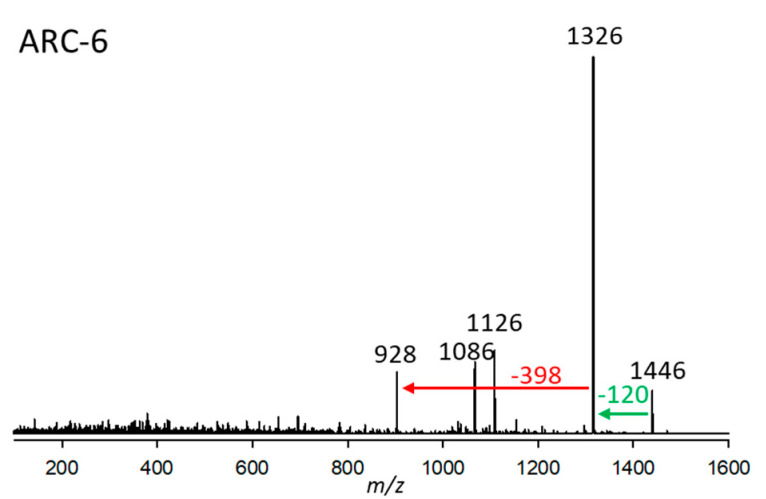
Collision-induced dissociation (CID) spectrum of ARC-6 (*m*/*z* 1446) from strain *A. carterae* CCMP122. The red arrow shows the neutral loss of 398 Da, indicative of the lipophilic arm (C1’–C22’) of ARC-6, while the green arrow shows the neutral loss of NaHSO_4_ (120 Da), forming the fragment *m*/*z* 1326, indicative of a sulfatation of ARC-6.

ARC-6, found in strain *A. carterae* CCMP122, had a pseudo-molecular ion of *m*/*z* 1446 and a retention time of 3.24 min. Like ARC-4, the CID spectrum of ARC-6 does not show many fragments (Figure 6). For this reason, the assignment of structural elements is hardly possible based on the present mass spectrometric data. However, ARC-4 and ARC-6 share a sulfatation, which can be deduced by the elimination of 120 Da. Furthermore, ARC-6 possesses a 6 Da heavier lipophilic arm (indicative of a triple saturation of the lipophilic arm) compared to ARC-4, indicated by the neutral loss of 398 Da from the desulfated fragment *m*/*z* 1278. The neutral loss of 398 Da is also observed in the CID spectrum of AM-B [12], indicating that AM-B and ARC-6 share the lipophilic arm.

#### 2.2.7. Variant ARC-7

**Figure 7:**
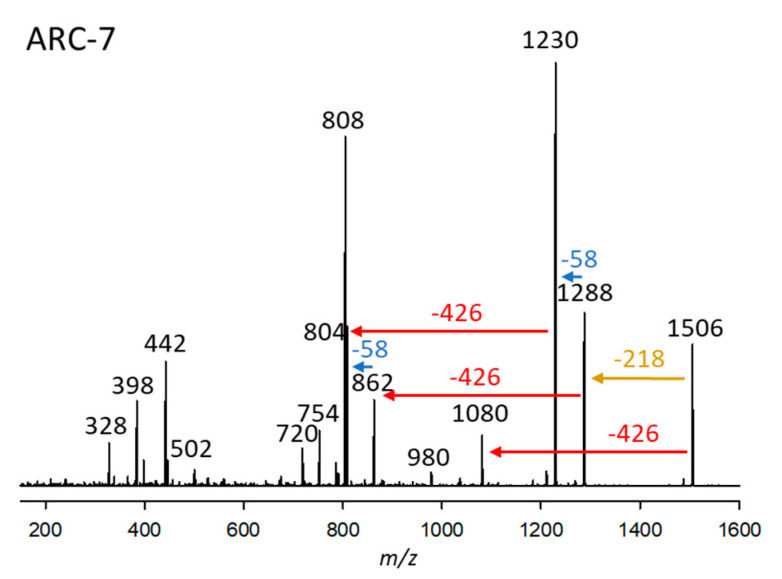
Collision-induced dissociation (CID) spectrum of ARC-7 (*m*/*z* 1506) from strain *A*. *carterae* ARC411. The red arrow shows the neutral loss of 426 Da, indicative of the lipophilic arm (C1’–C22’) of ARC-7. In comparison, the blue arrows indicate mass differences of 58 Da, consistent with a di-γ-hydroxy-carbonyl function of ARC-7. The brown arrow shows the elimination of the structural element between the end of the hydrophilic arm and the inner γ-hydroxy function.

ARC-7 was detected in *A*. *carterae* (strains ARC411, ARC412), *A*. *massartii* (strain ARC1342), and *A*. *tomasii* (strain ARC388). This variant had a retention time of 4.38 min and a pseudo-molecular ion of *m*/*z* 1506 (Figure 7). Like ARC-5 (Figure 5a), ARC-7 also belongs to the AM-18 type of AM with a 29,33-di-hydroxy-31-carbonyl group in the hydrophilic arm of the molecule (Figure 5a) indicated by the 58 Da mass differences between fragments *m*/*z* 1288/1230 and 862/804, respectively (Figure 5a and Figure 7). Another common structural feature among AM-18, ARC-5, and ARC-7 is indicated by the elimination of the neutral fragment 218 Da (Figure 5a and Figure 7) from the respective pseudo-molecular ions to the first fragment. This is indicative of a conserved structure among the three compounds between the carbonyl group and the end of the hydrophilic part (Figure 5a). On the other hand, ARC-7 shares the same lipophilic arm (C1’–C22’) with ARC-3 (Figure 3, Table S3) indicated by the neutral mass of 426 Da.

#### 2.2.8. Variant ARC-8

**Figure 8:**
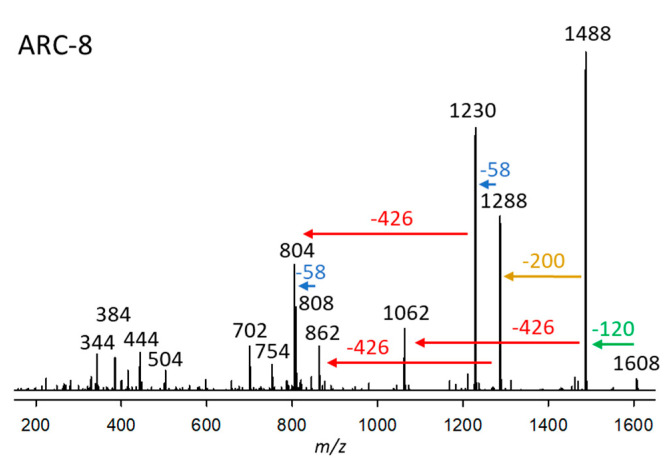
Collision-induced dissociation (CID) spectrum of ARC-8 (*m*/*z* 1608) from strains *A*. *carterae* ARC411. The green arrow indicates the neutral loss of NaHSO_4_ (120 Da), resulting in the formation of the fragment at *m*/*z* 1488, which is indicative of sulfatation of ARC-8. The red arrows show the neutral loss of 426 Da, indicative of the lipophilic arm (C1’–C22’) of ARC-8. In comparison, the blue arrows indicate mass differences of 58 Da, consistent with a di-γ-hydroxy-carbonyl function of ARC-8. The brown arrow shows the elimination of the structural element between the end of the hydrophilic arm (200 Da) and the inner γ-hydroxy function.

ARC-8, detected in strain *A. carterae* ARC411, had a retention time of 3.31 min and a pseudo-molecular ion of *m*/*z* 1608 (Figure 8). The fragmentation patterns of the CID spectra of ARC-7 were similar to ARC-8. Both variants were detected in the same strain (ARC411) and share the loss of the neutral fragment 426 Da of the lipophilic arm (C1’–C22’) and the 58 Da mass differences between fragments *m*/*z* 1506/1288/1230, and 1080/862/804, respectively (Figure 7 and Figure 8). However, an apparent difference between both CID spectra is the neutral loss of 218 Da from the pseudomolecular ion (*m*/*z* 1506) in the case of ARC-7 and of 200 Da from the [M-120]^+^ ion (*m*/*z* 1488) in the case of ARC-8. Interestingly, the same shift of the neutral loss of 218 to 200 Da is observed in the CID spectra of AM-18 and AM-19 (Figure 5a,b in Wellkamp et al. [12]). AM-18 and AM-19 are structurally identical, except that AM-19 has a sulfatation at C37 (carbon numbering according to Wellkamp et al. [12]). This analogy suggests that ARC-8 is a sulfated variant of ARC-7. This assumption is consistent with the observation that both compounds were detected in the same strain.

### 2.3. Diversity, Toxicity, and Amphidinol Profile of Strains

**Figure 9:**
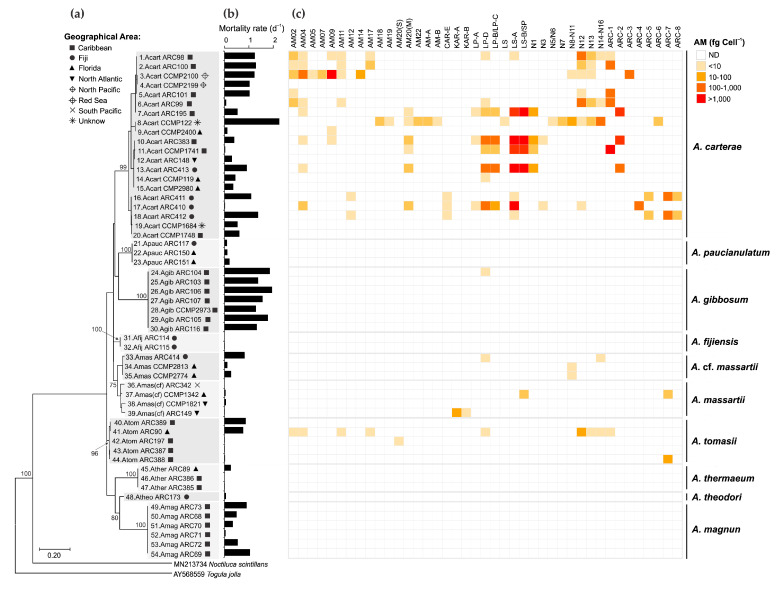
(**a**) Phylogenetic diversity of the 54 *Amphidinium* strains based on the maximal likelihood analysis of LSU rDNA gene related to (**b**) the toxicity of their crude methanol extract in the brine shrimp assay (mortality rate; d^−1^) and (**c**) concentration (fg cell^−1^) of amphidinol variants. AM = amphidinol, CAR = carteraol, KAR = karatungiols, LP = luteophanol, LS = lingshuiol, SP = symbiopolyol, N = unknown AM variants described by Wellkamp et al. [13], ARC = unknown variants described in this study; ND = Not detected.

Based on the maximal likelihood (ML) phylogenetic analysis of the LSU rDNA gene (Figure 9a), the 54 *Amphidinium* strains were distributed in ten species: *A*. *carterae* (20 strains), *A*. *fijiensis* (2 strains), *A*. *gibbosum* (7 strains), *A*. *magnum* (6 strains), *A*. *massartii* (4 strains), *A*. cf. *massartii* (3 strains), *A*. *paucianulatum* (3 strains), *A*. *theodori* (1 strain), *A*. *thermaeum* (3 strains), and *A*. *tomasii* (5 strains). Only *Amphinidum* strains used in this study were included in the ML analysis to show how they are genetically related to one another and AM content and brine shrimp mortality (see Karafas et al. [29] for a more comprehensive phylogeny of these strains, including sequences of other *Amphidinium* isolates). Most of the strains included in this study were isolated from the Caribbean (27 strains), Florida (10 strains), and Fiji (9 strains), although cultures from other locations were also obtained (i.e., North Atlantic, North Pacific, South Pacific, Red Sea; Figure 9a, Table S1). While *A. carterae* strains were present in all geographical locations, other species presented more restricted distributions, such as *A*. *massartii* (Fiji and Florida), *A*. cf. *massartii* (Fiji and Florida), and *A*. *tomasii* (Caribbean and Florida) (Figure 9a). The remaining species were reported in only one location: Caribbean (*A*. *gibbosum*, *A*. *magnum*), Fiji (*A*. *fijiensis*, *A*. *theodori*), and Florida (*A*. *thermaeum*).

The toxicity of the 54 *Amphidinum* strains was assessed using the brine shrimp assay [38]. Despite its lack of specificity, the broad sensitivity of the brine shrimp assay provides a valuable comparative measure of overall toxicity among diverse strains and species [39]. Different inter- and intraspecific patterns were observed in the toxicity of the strains based on the brine shrimp assay using crude methanol extracts (Figure 9b). While only strains with high toxicity (mortality rates = 1.2–2 d^−1^) were present in *A*. *gibbosum*, no or low toxicity was observed in *A*. *fijiensis*, *A*. *massartii*, *A*. *paucianulatum, A*. *theodori*, and *A*. *thermaeum* (mortality > 0.3 d^−1^) (Figure 9b). Strong intraspecific variability was observed in *A*. *carterae*, *A*. *magnum*, *A*. cf. *massartii*, and *A*. *tomasii,* which included strains showing very low to high mortality rates (0.02 to 2.25 d^−1^).

Total AM cell quotas found in this study ranged from non-detectable to a maximum of 4708 fg cell^−1^, with the highest levels of these compounds and broad diversity of AM variants found in *A. carterae* (Figure 9c). From the 20 *A. carterae* strains screened in this study, only three did not present detectable levels of any AM (strains ARC148, CCMP2199, CCMP2980). AM variants detected in high levels (>1000 fg cell^−1^) in *A*. *carterae* strains were AM-09 (strain CCMP2100), LS-A and LS-B/SP (both in strains ARC383 and ARC413), and ARC-1 (strain CCMP1741). Regarding the other species, only certain strains of *A*. *gibbossum*, *A*. *massartii*, *A*. cf. *massartii*, and *A*. *tomassi* showed low to moderate AM levels (2–93 fg cell^−1^) (Figure 9c).

**Figure 10:**
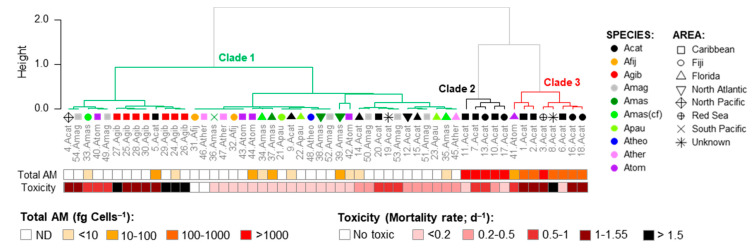
Clusters (C1-C5) of *Amphidinium* strains based on scores of the Principal Coordinate Analysis (PCoA) considering the composition of the AM variants (Figure 9) related to total AM cell quotas (fg Cell^−1^) and brine shrimp toxicity (mortality rate, d^−1^) of each strain. Acat = *A. carterae*, Afij = *A. fijiensis*, Agib = *A. gibbosum*, Amag = *A. magnum*, Amas = *A. massartii*, Amad(cf) = *A*. cf. *massartii*, Apauc = *A. paucianulatum,* Atheo = *A. theodori*, Ather = *A. thermaeum*, and Atom = *A. tomasii*. See Table S1 for the strain codes.

No clear geographical pattern was observed for the 54 strains regarding the brine shrimp toxicity (Figure S1a) and total AM cell quota (Figure S1b). Similarly, no correlation was observed between the two parameters (Figure S1c). A Principal Coordinate Analysis (PCoA; total variability = 58.89%) considering the composition of the AM variants, as well as total AM levels and the brine shrimp toxicity (Figure S2), indicated that the 54 *Amphidinium* strains were grouped in three main clusters (Figure 10). Cluster 1 included the majority of the strains with variable toxicity and AM levels varying from non-detectable to low (>10 fg cell^−1^) to moderate (10–1000 fg cell^−1^). AM variants in these clusters, when detected, were mainly AM20(S), KAR-B, and KAR-A. The other two clusters were composed almost exclusively of *A. carterae* strains with high AM levels (>1000 fg cell^−1^) and moderate to high toxicity (mortality rates of 0.4–1 and >1, respectively). These two clusters differentiated from one another mainly in their AM profiles: Cluster 2 was characterized by higher values of AM20(M), LP-A, LP-B/LP-C, LS-B/SP, N1, N3, ARC-2, and ARC-4, whereas Cluster 3 was characterized by AM02, AM04, AM05, AM07, AM11, AM12, AM14, AM17, AM18, AM19, AM-A, AM-B, LS, N5/N6, N7, N8-N11, N12, N13, N14-N16, ARC-3, ARC-5, ARC-7, ARC-8 (Figure 10 and Figure S2).

The lack of correlation between brine shrimp mortality and AM levels observed in this study may be related to the structural diversity of AM analogs, as previous studies have shown that some AM variants exhibit low hemolytic, antifungal, or cytotoxic bioactivity, despite being present at high concentrations [40,41,42,43]. Other bioactive compounds produced by certain *Amphidinium* strains, such as amphidinins [44] and amphirionin [45], may be the source of the observed toxicity in strains exhibiting high brine shrimp mortality but undetected AMs. Additionally, the genetic metabolic diversity commonly observed in dinoflagellates [46,47] could lead to the production of other compounds with synergistic effects that could mask or override the influence of AMs alone. These factors could collectively contribute to the complex and non-linear relationship observed between AM cell quotas and toxicity, emphasizing that AM levels alone are insufficient predictors of their biological and ecological impact. Overall, our results suggest that other uncharacterized compounds, besides AMs, are also responsible for fish kills associated with *Amphidinium* species in natural environments. Although some studies have shown allelopathic effects of certain AMs against benthic diatoms [48], the ecological roles of AMs are yet to be fully addressed. However, in structural terms, AMs are practically identical to karlotoxins, which have been hypothesized to be involved in grazing, defense, and prey capture [49]. Due to their structural similarity, comparable ecological functions can be assumed for AMs as well. It is also known that environmental factors, such as temperature and nutrient availability, may influence toxin production in dinoflagellates and partly explain the different AM cell quotas observed across strains from other locations and species.

## 3. Materials and Methods

### 3.1. Culturing and Phylogenetic Analysis of Amphidinium Strains

The 54 *Amphidinium* strains were obtained from the Algal Resources Collection (https://www.algalresourcescollection.com/, accessed on 23 June 2025) and the National Center for Marine Algae and Microbiota (https://ncma.bigelow.org/, accessed on 3 June 2025). Information on the origin is provided in Table S1. Strains were grown in 1.5-L duplicates (one for the brine shrimp assay and the other for the AM analysis) under a light:dark cycle of 14:10 h and a light intensity of ~50 μM photons m^−2^ s^−1^. Culture media, salinities, and temperatures used for the different strains are listed in Table S1.

The LSU rDNA sequences of *Amphidinium* strains (see Karafas et al. [29] for GenBank accession nos.) were aligned along with two outgroup sequences (GenBank accession nos. MN213734, AY568559) using the online software MAFFT 7 (https://mafft.cbrc.jp/alignment/server, accessed on 7 July 2025). The best nucleotide substitution model was determined using MEGA7 [50], and the Kimura-2-parameter model [51] was selected with invariant sites (K2 + I) and partial deletion of gaps. Maximum likelihood was measured using MEGA7, and the robustness of the inferred topology was supported by bootstrap resampling (1000 replicates).

### 3.2. Amphidinol Extraction

The solid pellets were poured into cryovials (Sarstedt, Nümbrecht, Germany), and the microtubes were rinsed with 1 mL of methanol (HPLC-grade, Merck, Darmstadt, Germany), which was added to the cryovial with the sample. Additionally, ~0.9 g of Lysing Matrix D (Thermo Scientific, Illkirch, France) was added to the sample. The cells were lysed by reciprocal shaking at a speed of 4.5 m s^−1^ for 45 s in a Bio 101 FastPrep Instrument (Thermo Savant, Illkirch, France). Subsequently, the vials were centrifuged for 15 min at 16,100× *g* (5415 R, Eppendorf, Hamburg, Germany), and 500 µL of the supernatant was transferred into a 0.45 µm spin filter (Merck Millipore, Darmstadt, Germany), which was centrifuged for 30 s at 2300× *g*. The filtrates were transferred into 2 mL HPLC crimp vials (Agilent Technologies). The remaining 500 µL of supernatant was placed into the previously used spin filter, which was centrifuged again for 30 s at 2300× *g*. Filtrates of the same sample were combined and left to dry overnight in the laboratory fume hood. Dry samples were reconstituted in methanol to a defined volume of 500 µL. Then, the vial was crimped with a silicone septum (11 mm Silver Aluminum Crimp Cap, PTFE/silicone septa, (Agilent Technologies, Waldbronn, Germany) and vortexed to ensure the complete solution of all sample constituents.

### 3.3. Analysis via Ultra-Performance Liquid Chromatography Coupled with Tandem Mass Spectrometry

An ultra-performance liquid chromatography (UPLC^®^) instrument coupled with tandem mass spectrometry (MS/MS) was used to identify and quantify the toxin levels of AMs as previously explained by Durán-Riveroll et al. [4]. The UPLC system included an ACQUITY UPLC column oven, an AQUITY UPLC I-class autosampler, and an ACQUITY UPLC I-class binary pump (Waters, Eschborn, Germany). The separation was achieved using Purospher^®^ STAR RP-18 endcapped (2 µm) Hibar^®^ HR 50-2.1 UHPLC column (Merck Millipore). A 0.5 µm OPTS-SOLV^®^ EXP™ precolumn (Sigma-Aldrich, Hamburg, Germany) was used to ensure the safety of the column. The entire system was coupled to an Xevo^®^ TQ-XS mass spectrometer (Waters). The software MassLynx (Version 4.2, Waters) was used to collect and analyze the data. Detection limits were defined as three times the signal-to-noise ratio (S/N), which were also directly calculated in TargetLynx XS. The implemented chromatography and mass spectrometry parameters for the UPLC-MS/MS measurements are listed in Table 2.

#### 3.3.1. Selected Reaction Monitoring (SRM) Mode

For the detection of known AMs and their quantification, the SRM mode was applied. Table S2 provides an overview of the transitions of every known AM, as well as for the AMs detected by Wellkamp et al. [12], named N1 to N16, and eight new variants described in this paper (ARC-1 to ARC-8).

The limits of detection (LoD) were calculated based on the signal-to-noise ratio (S/N) of the luteophanol-D (LPD) standard concentration expressed in ng µL^−1^. The LoD was expressed as fg sample^−1^. The concentration was calculated with Equation (1):

(1) $$\;LoD\;\left[fg\;{cell}^{-1}\right]=c\left(LPD\right)*\frac{3}{\frac{S}{N}\left(LPD\right)}\;*sample\;volume\;\left[µL\right]*\frac{1,000,000}{number\;of\;cells}$$

where LoD is the limit of detection, c(LPD) is the concentration of the luteophanol-D standard (ng µL^−1^), and S/N (LPD) is the signal-to-noise ratio of the luteophanol-D standard.

Identified AMs were calibrated against the LPD standard solution and expressed as LPD equivalents according to Equation (2):

(2) $$Toxin\left[ng{µL}^{-1}\right]=\frac{Peak\;Area\left(Toxin\right)*c\left(LPD\right)}{Peak\;Area\left(LPD\right)}$$

where Toxin is the concentration of measured toxin in ng µL^−1^, the Peak Area (Toxin) is the obtained peak area calculated by MassLynx from the toxin, c(LPD) is the concentration of the luteophanol-D standard, and the Peak Area (LPD) is the obtained peak area calculated by MassLynx from the LPD standard.

AMs were calibrated against an external LPD standard and expressed as LPD equivalents. Equation 3 shows the calculation of the cell quota in fg cell^−1^:

(3) $$Toxin\;cell\;quota\left[fg\;{cell}^{-1}\right]=\frac{Toxin[ng{µL}^{-1}]*Sample\;volume\left[µL\right]*1,000,000}{Number\;of\;cells}$$

where Toxin cell quota = concentration of toxin in fg per individual cell and Toxin = concentration of measured toxin in ng µL^−1^.

The LPD was isolated from the *Amphidinium carterae* strain ACRN03 [34]. Ten µg of the LPD was kindly provided by F. García-Camacho of the University of Almería, Spain. The powdery standard material was dissolved in 800 µL of methanol, resulting in an LDP concentration of about 13 ng µL^−1^. All quantitative values presented in this work are expressed as LPD equivalents and are therefore only semi-quantitative.

#### 3.3.2. Neutral Loss (NL) Measurement Mode

To date, fourteen neutral AM fragments have been identified as a result of the characteristic C1C1’ bond cleavage between the lipophilic and hydrophilic parts of AMs. Seven of them are non-sulfated neutral fragments, whilst the other seven are sulfated neutral fragments (Table S3). All strains of this study were screened in the neutral loss (NL) mode for each of these 14 neutral losses. Each strain was checked for peaks resulting from the NLs mentioned above. A signal-to-noise ratio of three was set as the minimum threshold for selecting peaks. The Q1-Masses of the detected peaks were further examined via the product ion spectra of the respective precursors.

#### 3.3.3. Full-Scan (FS) Measurement Mode

The full-scan measurements in the mass range from *m*/*z* 1000 to 1800 were used in addition to NL scans to ensure that potential AM variants would be detected, even if they did not form any of the known neutral losses listed in Table S3. Every peak that showed a retention time in the range of the known AMs, which is between 2.0 and 4.2 min, was checked for the presence of abundant Q1-Masses.

#### 3.3.4. Collision-Induced Dissociation (CID) Measurement Mode

Customized collision-induced dissociation experiments were generated for all *m*/*z* values detected by the NL and FS experiments for further investigation of the discovery of possible new AM variations. The starting mass stayed consistently at *m*/*z* 100 for every generated CID scan. The end mass had a value of about *m*/*z* 100 more than the recorded Q1-Mass. Ten thousand (10,000) was the set scanning rate of the mass spectrometer and was therefore kept consistent. The collision energy was consistently at 75 eV. The time frame of the CID scan was based on the respective retention time. The start was selected to be one minute earlier than the retention time, whilst the end was set to be one minute after the stated retention time. A time window of 2 min was therefore used in every CID scan. The flow state for liquid chromatography was set to start 0.01 min after the measurement began and was switched from the chromatography path to the waste path 0.01 min before the measurement window ended.

#### 3.3.5. Selection of Neutral Loss Scans

A common feature among all AMs is a bond cleavage between two vicinal hydroxyl groups near the tetrahydropyran ring B in the conserved central part of the molecules, separating AMs into the hydrophilic and lipophilic parts. With carbon numbering starting at the end of the molecule according to the IUPAC (Figure S4), the C numbers at the conserved cleavage site would depend on the carbon number of the hydrophilic parts of the molecules, and thus, result in different numbers across different AMs. To identify and visualize similarities and differences between lipophilic and hydrophilic parts of AMs, a numbering starting at both sides of the cleavage site has been proposed, where C1’ to C n’ denominates the lipophilic part and C1 to Cn the hydrophilic part of AMs [Wellkamp et al. [12] (Figure S4).

### 3.4. Brine Shrimp Assay

Pellets were diluted in 10 mL aqueous methanol (MeOH/H_2_O: 80/20 (*v*/*v*)) and sonicated for 1 min, and left to extract overnight. The obtained extract was filtered through glass-fiber filters (Whatman GF/F) and then dried using a rotary evaporator (Rotavap). The solution was weighed and then diluted in DMSO to achieve a final concentration of 100 µg mL^−1^. Brine shrimp (*Artemia salina*) cysts were incubated in seawater (36 salinity) with aeration at room temperature (~20 °C) for 3 days and used in a toxicity assay following a modification of the protocol proposed by Lincoln et al. [38]. Briefly, brine shrimp nauplii were collected using a pipette by attracting them to the surface with proximity illumination, and their concentration was adjusted to ~30 individuals mL^−1^. For each *Amphidinium* strain, 2 mL of the Brine shrimp sample was added to 9 wells of a 24-well plate to obtain ~60 individuals per well. Three of these wells were inoculated with 25 µL of the culture extract to get a final concentration of 1.2 µL mL^−1^ (1.25% DMSO), and six wells were used as controls (three wells with 25 µL of DMSO and the other three with 25 µL of seawater). After 30 min, the bottom of each well was examined with an inverted microscope to check for deaths due to inoculation (i.e., initial mortality). Plates were then examined after 24 h and 48 h to count dead individuals. After that, a drop of Lugol’s solution was added to each well to determine the total number of individuals. The obtained mortalities at 24 h and 48 h were adjusted by subtracting the initial mortality and the number of dead individuals in the controls. The mortality rates (deaths d^−1^) were estimated using Equation (4):

(4) $$mortality\;rate\;\left(d^{-1}\right)=\;\frac{LN(number\;dead\;individuals\;after\;48h)}{48h}\;\times 24h$$

### 3.5. Statistical Analysis

We performed a Principal Coordinate Analysis (PCoA) to assess whether AM composition was related to Total AM levels and brine shrimp mortality using the function ‘cmdscale’ of the package vegan of the software R 4.3.2. As AM cell quotas (both total and for the different variants) and mortality rates are expressed in different orders of magnitude, the values of these variables for each strain were previously scaled from 0 to 1 and then log transformed [ln(x + 1)] (*n* = 54). PCoA scores were then included in a cluster analysis using the ‘hclust’ function from the base R package to determine groups of strains.

## 4. Conclusions

This study presents, for the first time, the characterization of amphidinols (AMs) in a comprehensive number of *Amphidinium* strains from several species, obtained from diverse geographical areas. Total AM cell quotas varied widely: from undetectable levels to over 4700 fg per cell. However, no clear correlation emerged between Total AM levels and brine shrimp toxicity levels. The multivariate clustering analyses further indicated that strain grouping was predominantly influenced by complex AM profiles, with high-AM, high-toxicity clusters essentially consisting of *A. carterae* strains. Yet, these did not universally correlate with toxicity. Overall, these findings underscore the complexity of AM production and toxicity in *Amphidinium*, suggesting that multiple factors, including strain-specific biosynthetic capacities and the presence of other bioactive compounds, contribute to the observed biological effects, rather than AM levels alone. Future work is needed to characterize novel AMs structurally using NMR spectroscopy. Furthermore, to gain insight into the ecological functions of AMs, additional research is required in the form of co-cultivation experiments with various protistan species or potential *Amphidinium* grazers, as well as with different nutrient, temperature, and light regimes. In this context, investigating whether environmental stimuli can trigger AM expression may contribute to the understanding of the ecological relevance of these compounds, their mitigation, or, given their biological activities and pharmacological application, their potential for large-scale production.

## Figures and Tables

**Table 1:** Confirmed new AM variants, their retention times, Q-1 Masses, detection mode, and operational names. See Table S1 for information on the *Amphidinium* strains.

Variant Name	Strain Code	Strain Name	*t_R_* (min)	Q1-Mass (*m*/*z*)	Detection Mode
ARC-1	1.Acart	ARC101	3.89	1226	Neutral loss
ARC-2	10.Acart	ARC383	3.03	1266	SRM
ARC-3	3.Acart	CCMP2100	2.90	1358	Neutral loss
ARC-4	17.Acart	ARC410	2.93	1398	SRM
ARC-5	19.Acart	ARC412	3.87	1426	SRM
ARC-6	8.Acart	CCMP122	3.20	1446	Neutral loss
ARC-7	16.Acart	ARC411	4.35	1506	Full scan
ARC-8	16.Acart	ARC411	3.26	1608	Full scan

**Table 2:** Chromatographic and mass spectrometric parameters for the measurement of AMs for all three measurement modes (NL, FS, CID).

Chromatographic parameters	
Eluent composition	A: 500 mL ultrapure water + 252.5 µL NH_4_OH (25%) B: 450 mL acetonitrile + 50 mL ultrapure water + 252.5 µL NH_4_OH (25%)
Eluent gradient	From 80% Eluent A to 10% Eluent A
Total duration (min)	5
Flow rate (mL min^−1^)	0.2
Injection volume (µL)	0.5
Collision energy (eV)	75 85 for AMs over 1500 *m*/*z* during product ion scans
Scanning time (sec)	0.133
Scanning rate (points per peak)	12
**Spectrometric parameters**	
Ion source	
Capillary voltage (kV) Cone voltage (V) Desolvation temperature (°C)	3.00 40 600
Gas flow	
Desolvation gas flow (L h^−1^) Cone gas flow (L h^−1^) Nebulizer gas flow (bar)	1000 150 7.0
Further settings	
Autosampler temperature (°C) Column temperature (°C) Electrospray Full-scan mass range (*m*/*z*)	10 40 ES+ 1000–1800

## Data Availability

The data presented in this study are available in this article and Supplementary Material.

## Supplementary Materials

The following supporting information can be downloaded at: https://www.mdpi.com/article/10.3390/md23090332/s1, Figure S1: (a) Mortality rate (d^−1^) and (b) total amphidinol (AM) cell quotas (fg Cell^−1^) of *Amphidinium* strains per geographical areas. (c) Relationship between Mortality rate (d^−1^) and (b) total amphidinol (AM) levels (fg Cell^−1^); Figure S2: Principal Coordinate Analysis (PCoA) considering the composition of the AM variants, as well as total AM levels and brine shrimp toxicity; Figure S3: Original carbon numbering of AM21 by Satake et al. [16]; Figure S4: Fragmentation scheme of AM21 [13] and modified carbon numbering. The C-1’/C-1 bond cleavage site is marked in the red frame; Table S1. Collecting information and culturing conditions of the Amphidinium strains. * Strain codes in Karafas et al. [31]. Sal = Salinity, Temp = Temperature; Table S2: Overview of known AM transitions. AM = amphidinol; AMD = amdigenol; CAR = carteraol; KAR = karatungiol; LP = luteophanol; LS = lingshuiol; SP = symbiopolyol; unknown AM variants named N1-N16 described by Wellkamp et al. [13]. The remaining unknown AM variants, named ARC-1 to ARC-8, are described in this work. * Molina et al. [37]; ** Satake et al. [16]; Table S3. Detected neutral losses (NLs) and corresponding Q1-Masses found in various Amphidinium strains. Table S4. Additional Q1-Masses detected through the full-scan measurement mode (FS).

## Abbreviations

The following abbreviations are used in this manuscript:

HAB	Harmful Algal Blooms
AM	Amphidinols
LP	Luteophanols
LS	Lingshuiols
SP	Symbiopolyols
KAR	Karatungiols
CAR	Carteraols
LC-MS/MS	Liquid chromatography tandem mass spectrometry
UHPLC-MS/MS	Ultra-high performance liquid chromatography tandem mass spectrometry
SRM	Selected reaction monitoring
NMR	Nuclear magnetic resonance spectroscopy
MS	Mass spectometry
NL	Neutral loss
NL	Neutral mode
FS	Full scan
CID	Collision induced dissociation
ESI	Electrospray ionization
ARC	Algal Resources Collection
