# Supplementary material for: High Inter- and Intraspecific Variability in Amphidinol Content and Toxicity of Amphidinium Strains
Source: Mar Drugs. 2025 Aug 22;23(9):332. doi: 10.3390/md23090332 (PMC12471367; doi:10.3390/md23090332)
Supplement: Supplementary file 1 [file marinedrugs-23-00332-s001.zip › marinedrugs-3750973-supplementary.pdf]

Figure S1

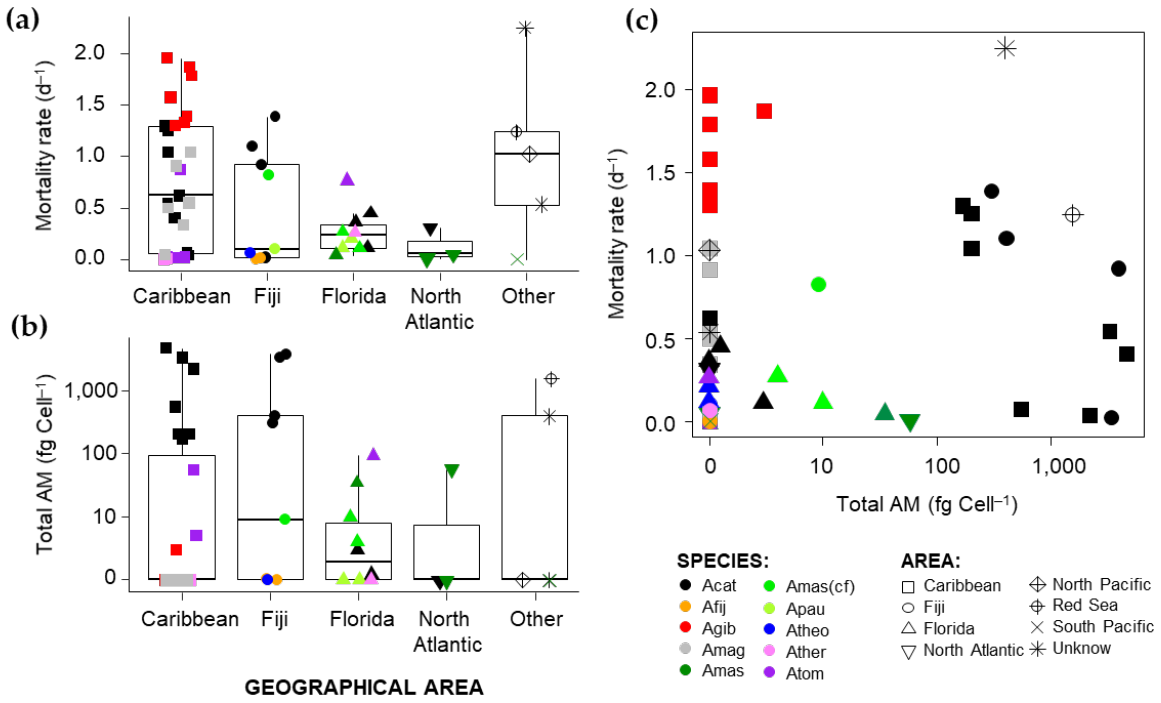

Figure S1. (**a**) Mortality rate (d⁻¹) and (**b**) total amphidinol (AM) cell quotas (fg Cell⁻¹) of *Amphidinium* strains per geographical areas. (**c**) Relationship between mortality rate (d⁻¹) and (b) total amphidinol (AM) levels (fg Cell⁻¹).

Figure S2

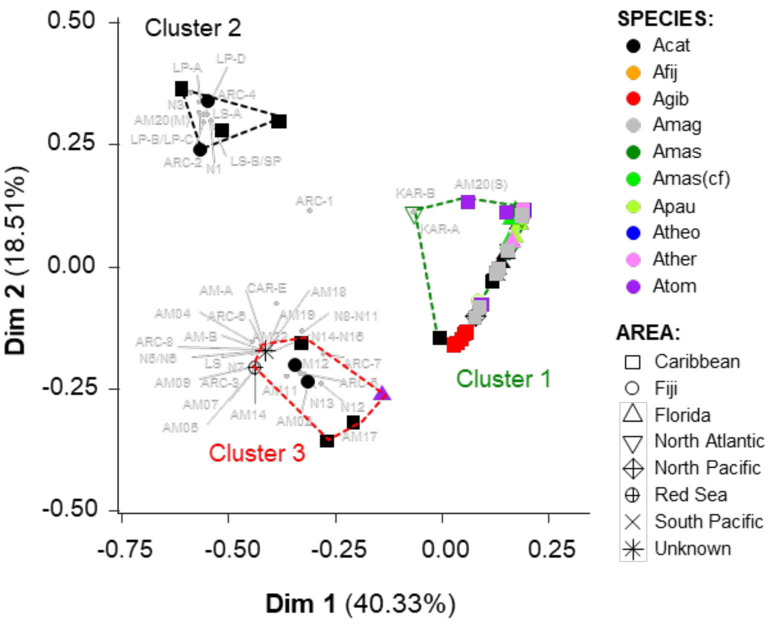

Figure S2. Principal Coordinate Analysis (PCoA) considering the composition of the AM variants, as well as total AM levels and brine shrimp toxicity.

Figure S3

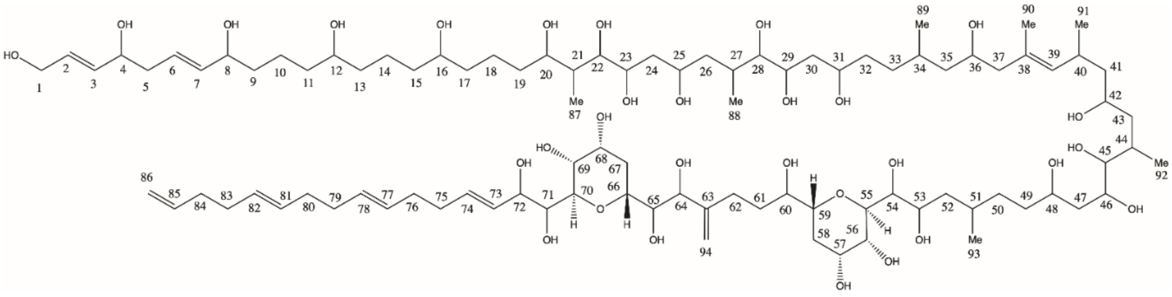

Figure S3. Original carbon numbering of AM21 by Satake et al. [16].

Figure S4

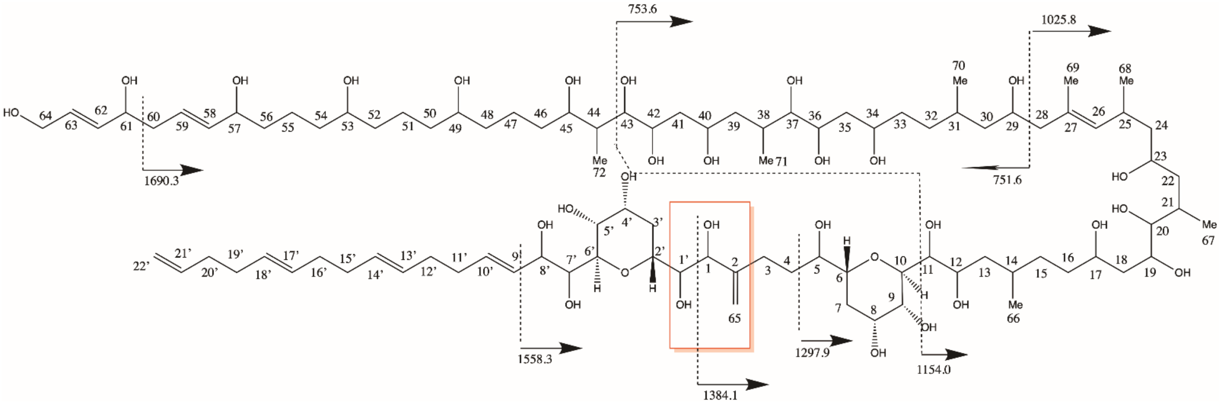

Figure S4. Fragmentation scheme of AM21 [13] and modified carbon numbering. The C-1'/C-1 bond cleavage site is marked in the red frame.

Table S1. Collecting information and culturing conditions of the *Amphidinium* strains. *Strain codes in Karafas et al. [31]. Sal = Salinity, Temp = Temperature.

Species	Strain Code	Strain Name	Other Strain Name*	Geographical Area	Collection Site	Collection Year	Culture conditions	
							Media/Sal	Temp (°C)
A. carterae	1.Acat	ARC98	Amca0406-2	Caribbean	Little San Salvador Island, Bahamas	2004	L2 39	20
	2.Acat	ARC100	Amca0406-4	Caribbean	Little San Salvador Island, Bahamas	2004	L2 39	20
	3.Acat	CCMP2100		Red Sea	Eilat, Israel	2001	L1 36	20
	4.Acat	CCMP2199		North Pacific	Aqua Hediona Lagoon, California, USA	2002	L1 36	20
	5.Acat	ARC101	Amca0406-5	Caribbean	Little San Salvador Island, Bahamas	2004	L2 39	20
	6.Acat	ARC99	Amca0406-3	Caribbean	Little San Salvador Island, Bahamas	2004	L2 39	20
	7.Acat	ARC195	Amca0812-1	Caribbean	La Parguera, Puerto Rico	2008	L1 36	20
	8.Acat	CCMP122		Unknown	Unknow	1980	L1 36	20
	9.Acat	CCMP2400		Florida	New Pass Channel, Florida, USA	2004	L1 36	20
	10.Acat	ARC383	Amca1412-2	Caribbean	Bahia Cofresi, Dominican Republic	2014	L1 39	20
	11.Acat	CCMP1741		Caribbean	Elbow Cay, Belize	1996	L1 36	20
	12.Acat	ARC148		North Atlantic	Offshore North Carolina, USA	Unknow	L2 39	20
	13.Acat	ARC413	Amca1506-4	Fiji	Korotoga Fiji	2015	L1 36	25
	14.Acat	CCMP119		Florida	Knight Key, Florida, USA	1986	L1 36	20
	15.Acat	CCMP2980		Florida	Marquesas Keys, Florida, USA	2009	L1 36	20
	16.Acat	ARC411	Amca1506-2	Fiji	Korotoga, Fiji	2015	L1 39	25
	17.Acat	ARC410	Amca1506-1	Fiji	Korotoga, Fiji	2015	L1 39	25
	18.Acat	ARC412	Amca1506-3	Fiji	Korotoga, Fiji	2015	L1 36	25
	19.Acat	CCMP1684		Unknown	Unknow	Unknow	L1 36	20
	20.Acat	CCMP1748		Caribbean	Main Lagoon, Belize	1997	L1 36	20
A. paucianulatum	21.Apauc	ARC117	Ampa0508-3	Fiji	Korotoga, Fiji	2005	K 39	20
	22.Apauc	ARC150	Ampa0606-2	Florida	Palma Sola Causeway, Florida	2006	K 39	20
	23.Apauc	ARC151	Ampa0606-3	Florida	Palma Sola Causeway, Florida	2006	K 39	20
A. gibbosum	24.Agib	ARC104	Amgi0406-1	Caribbean	Little San Salvador Island, Bahamas	2004	L2 39	20
	25.Agib	ARC103		Caribbean	Little San Salvador Island, Bahamas	Unknow	L2 39	20
	26.Agib	ARC106	Amgi0406-3	Caribbean	Little San Salvador Island, Bahamas	2004	L2 39	20
	27.Agib	ARC107		Caribbean	Little San Salvador Island, Bahamas	2004	L2 39	20
	28.Agib	CCMP2973		Caribbean	South of Brewers Bay Beach, U.S. Virgin Islands	2008	L2 39	20
	29.Agib	ARC105	Amgi0406-2	Caribbean	Little San Salvador Island, Bahamas	2004	F/2 36	20
	30.Agib	ARC116	Amgi0508-1	Caribbean	La Parguera, Puerto Rico	2005	L2 39	20
A. fijiensis	31.Afij	ARC114	Amfi0508-1	Fiji	Korotoga, Fiji	2005	L2/K 39	20
	32.Afij	ARC115	Amfi0508-2	Fiji	Korotoga, Fiji	2005	L2/K 39	20
A. cf. massartii	33.Amas(cf)	ARC414	Amma1506-1	Fiji	Korotoga, Fiji	2015	L1 39	25
	34.Amas(cf)	CCPM2774		Florida	Marquesas Keys, Florida, USA	2006	L1 36	20
	35.Amas(cf)	CCPM2813		Florida	Marquesas Keys, Florida, USA	1900	L1 36	20
A. massartii	36.Amas	ARC342	Amma1208-1	South Pacific	Palmyra Atoll	2012	L2 39	20
	37.Amas	CCMP1342		Florida	Knight Key, Florida, USA	1991	L1 36	20
	38.Amas	CCMP1821		North Atlantic	Succotash Marsh, Rhode Island, USA	1997	L1 36	20
	39.Amas	ARC149	Amma0607-1	North Atlantic	Offshore North Carolina, Gulf Stream	2006	L2 39	20

Table S1. (continuation)

Species	Strain Code	Strain Name	Other Strain Name*	Geographical Area	Collection Site	Collection Year	Culture conditions	
							Media/Sal	Temp (°C)
A. tomasii	40.Atom	ARC389	Amto1412-3	Caribbean	Bahia Cofresi, Dominican Republic	2014	L1 39	20
	41.Atom	ARC90	Amto0304-2	Florida	Coast of Florida, USA	2003	L1 36	20
	42.Atom	ARC197	Amto0812-2	Caribbean	Bahia Phosphorescent Bay, Pueto Rico	2008	L1 36	25
	43.Atom	ARC387	Amto1412-1	Caribbean	Bahia Cofresi, Dominican Republic	2014	L1 39	20
	44.Atom	ARC388	Amto1412-2	Caribbean	Bahia Cofresi, Dominican Republic	2014	L1 39	20
A. thermaeum	45.Ather	ARC89	Amth0304-1	Florida	Siesta Key, Florida, USA	2003	K 39	20
A. theodori	46.Ather	ARC386	Amth1412-2	Caribbean	Bahia Cofresi, Dominican Republic	2014	L1 39	20
	47.Ather	ARC385	Amth1412-1	Caribbean	Bahia Cofresi, Dominican Republic	2014	L1 39	20
	48.Atheo	ARC173	Amth0702-1	Fiji	Korotoga, Fiji	2007	K 39	20
A. magnum	49.Amag	ARC73	Amma0206-6	Caribbean	Grand Bahama Beach, Bahamas	2002	L2 39	20
	50.Amag	ARC68	Amma0206-5	Caribbean	Grand Bahama Beach, Bahamas	2002	K 39	20
	51.Amag	ARC70	Amma0206-2	Caribbean	Grand Bahama Beach, Bahamas	2002	K 39	20
	52.Amag	ARC71	Amma0206-3	Caribbean	Grand Bahama Beach, Bahamas	2002	K 39	20
	53.Amag	ARC72	Amma0206-4	Caribbean	Grand Bahama Beach, Bahamas	2002	K 39	20
	54.Amag	ARC69	Amma0206-1	Caribbean	Grand Bahama Beach, Bahamas	2002	L2 39	20

Table S2. Overview of known AM transitions. AM = amphidinol; AMD = amdigenol; CAR = carteraol; KAR = karatungiol; LP = luteophanol; LS = lingshuiol; SP = symbiopolyol; unknown AM variants named N1-N16 described by Wellkamp et al. [13]. The remaining unknown AM variants, named ARC-1 to ARC-8, are described in this work. * Molina et al. [37] ; ** Satake et al. [16] .

Toxin	Q1-Mass (*m/z*)	Q3-Mass (*m/z*)	Toxin	Q1-Mass (*m/z*)	Q3-Mass (*m/z*)
AM1	1512	974	LP-D	1330	904
AM2	1398	1006	LS	1374	976
AM3	1350	932	LS-A	1296	904
AM4	1324	932	LS-B	1266	754
AM5	1394	976	SP	1266	754
AM6	1368	976	N1	1268	876
AM7	1254	742	N2	1430	1038
AM9	1350	932	N3	1458	1066
AM10	1296	904	N4	1316	876
AM11	1500	988	N5	1346	946
AM12	1426	914	N6	1346	946
AM13	1452	914	N7	1346	928
AM14	1288	742	N8	1364	946
AM15	1186	760	N9	1364	946
AM17	1306	816	N10	1364	946
AM18	1382	964	N11	1364	946
AM19	1484	946	N12	1326	946
AM20 (M)*	1346	904	N13	1326	928
AM20 (S)**	1653	1260	N14	1344	946
AM21	1798	1406	N15	1344	946
AM22	1668	1330	N16	1344	946
AM-A	1362	964	ARC-1	1226	834
AM-B	1464	946	ARC-2	1266	874
AMD-G	1300	768	ARC-3	1358	932
CAR-E	1422	1030	ARC4-	1398	1278
KAR-A	1480	1082	ARC-5	1426	1150
KAR-B	1462	1064	ARC-6	1446	1326
LP-A	1278	754	ARC-7	1506	1230
LP-B	1344	904	ARC-8	1608	1488
LP-C	1344	904			

Table S3. Detected neutral losses (NLs) and corresponding Q1-Masses found in various *Amphidinium* strains.

Strain code	Strain name	t_R (min)	Q1-Mass (m/z)	Neutral Loss
3.Acart	CCMP2100	2.90	1358	426
3.Acart	CCMP2100	2.95	1360	426
5.Acart	ARC101	3.89	1226	398
5.Acart	ARC101	2.94	1328	392/512
6.Acart	ARC99	3.79	1725	398
8.Acart	CCMP122	3.20	1446	518
16.Acart	ARC411	3.39	1627	338
17.Acart	ARC410	2.93	1398	338/512
20.Acart	CCMP1748	3.09	1329	562
21.Amas(cf)	ARC117	3.32	1105	418
33.Amas(cf)	CCMP2774	3.33	1152	398
38.Amas	CCMP1821	3.81	1420	418
38.Amas	CCMP1821	3.84	1492	398

Table S4. Additional Q1-Masses detected through the full-scan measurement mode (FS).

Strain Code	Strain Name	t_R (min)	Q1-Mass (m/z)
4.Acar	CCMP2199	4.4	1022
5.Acar	ARC101	4.15	1061
5.Acar	ARC101	3.4	1272
10.Acar	ARC383	3.21	1281
16.Acar	ARC411	3.06	1068
16.Acar	ARC411	2.74	1119
16.Acar	ARC411	4.35	1506
16.Acar	ARC411	3.26	1608
17.Acar	ARC410	3.53	1378
18.Acar	ARC412	3.73	1017
20.Acart	CCMP1748	3.77	1180
22.Apauc	ARC150	3.1	1126
23.Apauc	ARC151	2.57	1115
25.Agib	ARC103	4.58	1411
27.Agib	ARC107	2.89	1214
28.Agib	CCMP2973	4.12	1230
32.Afij	ARC115	2.88	1304
34.Amar(cf)	CCMP2774	3.48	1386
34.Amas(cf)	CCMP2774	2.89	1046
35.Amas(cf)	CCMP2813	2.88	1090
37.Amas	CCMP1342	2.9	1626
39.Amas	ARC149	3.99	1478
42.Atom	ARC197	2.76	1092
43.Atom	ARC387	3.66	1730
44.Atom	ARC388	3.39	1032
44.Atom	ARC388	3.75	1650
45.Ather	ARC89	3.27	1127
48.Atheo	ARC173	4.22	1016
48.Atheo	ARC173	3.19	1033
